# Parenting Interventions for Children with Tic Disorders: Professionals’ Perspectives

**DOI:** 10.1007/s10826-015-0317-1

**Published:** 2015-11-14

**Authors:** Gemma A. L. Evans, Anja Wittkowski, Hannah Butler, Tammy Hedderly, Penny Bunton

**Affiliations:** School of Psychological Sciences, University of Manchester, 2nd Floor Zochonis Building, Manchester, M13 9PL UK; Evelina London Children’s Hospital, St Thomas’ Hospital, London, SE1 7EH UK

**Keywords:** Tourette syndrome, Tic disorders, Parenting, Parent intervention, Professionals

## Abstract

Tic disorders can have an emotional and social impact on children and families, which can in turn have a reciprocal impact on tics. Research into parenting interventions within this population is limited. Twenty-five professionals’ views on the acceptability, effectiveness, feasibility and utility of parenting interventions were explored using Q-methodology. Three highly correlated factors emerged, indicating three viewpoints with discrete elements that were underpinned by similar general perspectives. All factors endorsed a psychological approach, the importance of parenting practices, and theoretical and clinical justifications for parenting interventions. Discrete elements of the viewpoints debated the advocated focus, barriers and audience of interventions. Multidisciplinary professionals endorsed parenting interventions as a therapeutic tool within tic disorders. Results provide suggestions to further develop and implement interventions.

## Introduction

Tics are recurrent, non-rhythmic, motor movements or vocalisations. Tics themselves are relatively common and are mildly and transiently experienced by around 10 % of children (Verdellen et al. 2011). Tics are, however, also characteristic of tic disorders. Prevalence across tic disorders varies from 0.77 % for Tourette syndrome to 2.99 % for transient tic disorder (Knight et al. 2012). Co-morbid conditions occur in around 90 % of individuals (Robertson and Cavanna 2008), and include attention deficit hyperactivity disorder (ADHD), obsessive compulsive behaviours, depression, anxiety, conduct difficulties, autism and learning difficulties (Robertson 2000; Robertson and Cavanna 2008).

Tic disorders and co-morbidities significantly affect children and families. Children may experience social, cognitive and emotional difficulties (Robertson and Cavanna 2008; Storch et al. 2007), while parents may experience increased stress and negative life events (Cooper et al. 2003; Robertson and Cavanna 2008). Furthermore, environmental, social and emotional factors can influence tic severity (Robertson and Cavanna 2008), thus, parental management and problematic family functioning may inadvertently contribute to tic exacerbation, which may heighten familial stress; creating a reciprocal cycle.

First-line recommended psychosocial interventions for tic disorders include child-directed behavioural interventions, namely those using habit reversal or exposure with response prevention techniques (Verdellen et al. 2011). However, family interventions also seem justified given the familial implications. Despite recognition of the importance of family education and support (Verdellen et al. 2011), this area is under-researched. Within a randomized controlled trial (RCT) design, only one study by Scahill et al. (2006) evaluated parent training for children with tic disorders and conduct difficulties. Although tics did not reduce, effects on disruptive behaviour were promising, indicating potential valuable clinical utility for co-morbid difficulties.

The effectiveness of parent training has been demonstrated across a number of neurodevelopmental disorders. For example, RCT studies have shown positive effects of parent-based interventions for children with intellectual disabilities/developmental delay (Leung et al. 2013; McIntyre 2008; Plant and Sanders 2007; Roberts et al. 2006; Roux et al. 2013), autistic spectrum conditions (Sofronoff et al. 2004; Whittingham et al. 2009) and attention deficit disorder (Azevedo et al. 2013; Hoath and Sanders 2002; Jones et al. 2007). Whilst these interventions have been directed at parents, some studies have also evaluated treatment models in which adjunctive parent interventions are implemented alongside child-directed treatments (e.g., Autistic spectrum conditions: Sofronoff et al. 2007; ADHD: Webster-Stratton et al. 2011). Indeed, within tic disorders there have been some attempts to incorporate parent-directed elements into child-focused interventions. For example, Piacentini et al. (2010) evaluated the use of a ‘comprehensive behavioural intervention for tics (CBIT)’ in a large RCT involving children and adolescents. The CBIT treatment involved aspects of habit reversal training, relaxation training and a functional intervention. Although treatment was predominantly child-focused, parents were included for all or parts of sessions and results showed positive effects of treatment on tic severity and tic-related impairment. Similarly, McGuire et al. (2015) implemented a RCT of a modularized cognitive behavioural intervention termed ‘living with tics’. The intervention involved both youths and parents, with specific parent-training modules, and results showed positive impacts on child quality of life and tic impairment. Incorporating both child and parent based elements into treatment, however, means that the factors of causation of change are difficult to establish, particularly in the context of such limited investigations into parent interventions. Furthermore, the potential for parent-only interventions is important in clinical practice, given that there may be limitations in the extent of involvement of children in treatment (e.g., due to age, developmental ability, co-morbid difficulties, willingness). Thus, establishing the potential intervention possibilities for parent-only interventions offers value.

Within the development of treatment interventions, preliminary exploration of relevant components and potential barriers is important and may be achieved using qualitative methodologies (Campbell et al. 2000). Exploring the views of professionals who have clinical experience in administering such interventions or experience of working with intended treatment populations may thus provide crucial information in the initial stages of intervention design and evaluation. Exploration of professionals’ views during these early stages of treatment development and implementation has been achieved using a number of methods, including Q-methodology. Q-methodology permits exploration of subjective viewpoints in a reliable, experimental and quantifiable manner (Watts and Stenner 2012) and is being increasingly used within healthcare research to explore staff and patients opinions of interventions (e.g., Absalom-Hornby et al. 2012; Butler et al. 2014; Westbrook et al. 2013).

The current study thus aimed to explore professionals’ views of parenting interventions within tic disorders using Q-methodology for the first time, with particular consideration to perceived acceptability, feasibility, effectiveness and utility.

## Method

### Design

The study used Q-methodology, whereby participants systematically rank statements according to agreement. The relative ranked positions reflect emergent viewpoints, permitting a reliable and quantifiable means of exploring participant opinion (Watts and Stenner 2012). This study had full ethical approval.

### Participants

Participants were recruited via email and web-based advertisements. The project was advertised through a tic disorders charity, specialist tic disorders service and a paediatric interest mailing group. Participants with experience of working professionally with tic disorders and/or delivering parenting interventions were included. No exclusion criteria were applied.

### Q-Methodology Procedure

#### Q-Set Development

The statements that are systematically ranked in Q-methodology are termed the Q-set. Information to develop the Q-set was derived from various sources (Watts and Stenner 2012). Academic literature, television shows and websites were searched and interviews were completed with parents of children with tic disorders who had previously participated in a pilot parenting group at one of the study recruitment sites. Themes were extracted and representative statements generated (n = 244) which were reviewed and refined by the research team to produce 73 final statements. These were considered to offer a balanced and representative coverage of opinions (Watts and Stenner 2012).

#### Data Collection

**Fig. 1:**
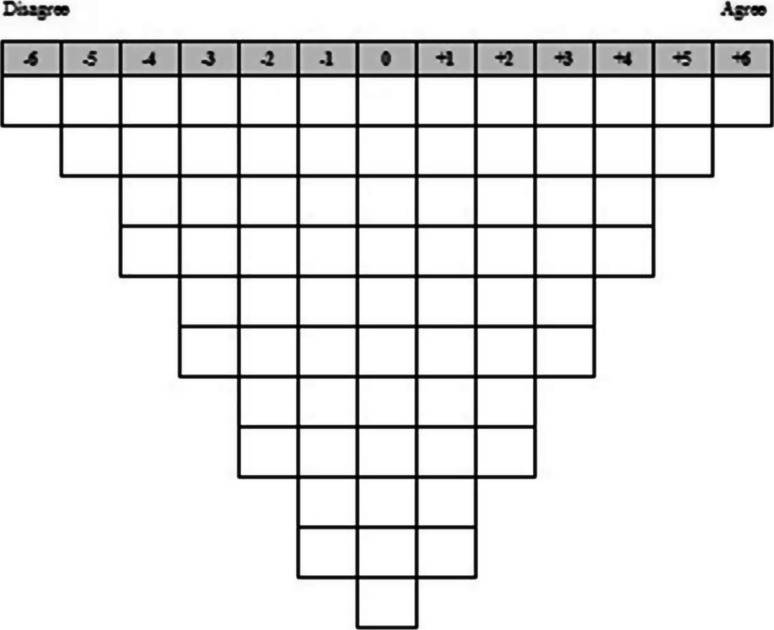
Q-sort grid

Q-sorts were completed via a secure website link. Participants firstly categorised the 73 statements as agree, neutral or disagree and then ranked statements from most agree (+6) to most disagree (−6), using a forced choice distribution grid (Fig. 1) . Free-text, post-sort questions then elicited further information about the statements ranked at the extreme ends of the Q-sort grid, as well as general views about parenting interventions in tic populations.

#### Data Analysis

Factor analysis was undertaken using PQMethod (Schmolck and Atkinson 2012). Q-methodology involves by-person factor analysis (Watts & Stenner 2012), identifying factors upon which participants load due to similar sorting patterns (Watts and Stenner 2005). A principal components factor analysis was conducted: factors with an eigenvalue >1 were extracted and subjected to varimax rotation. Q-sorts with significant factor loadings were merged using a weighted averaging procedure to create a factor array, or representative sorting pattern. Significant factor loadings can be determined using several criteria (Watts and Stenner 2012). Commonly, a *p* < 0.01 threshold is used, however, should this result in multiple confounding Q-sorts (which load significantly onto more than one factor and are excluded from analyses), increasing the loading stringency by raising the significance threshold is recommended (Watts and Stenner 2005).

Factors were interpreted using factor arrays, demographic information and post-sort questions. Factor-specific views were identified using statements that were statistically distinguishing (*p* < 0.01) or assigned distinctively extreme ratings compared to other factors. Shared views were explored using consensus statements and statements placed at extreme rankings across all factors.

## Results

### Participant Information

Twenty-five professionals participated, a sufficient sample size for Q-methodology (Watts and Stenner 2012). Table 1 presents demographic characteristics.

**Table 1:** Participant demographic information

Question	Number
Professional role	
Charity Worker	3
Clinical Psychologist	7
Research Assistant Psychologist	1
Medic	1
Paediatric Neurologist	1
Psychiatrist	2
Trainee Clinical Psychologist	8
Primary Mental Health Worker	1
Trainee Psychotherapist	1
Worked professionally with children and parents	
Yes	25
No	0
Professional involvement in parenting groups/interventions	
Yes	20
No	5
Frequency of involvement (if answered yes)	
Very frequently	6
Frequently	8
Occasionally	5
Rarely	1
Professional involvement with children with tic disorders and parents	
Yes	17
No	8
Frequency of involvement (if answered yes)	
Very frequently	4
Frequently	7
Occasionally	6
Rarely	0

### Q-sort Analysis

Three factors were extracted and rotated, accounting for 68 % of study variance. Twenty-two Q-sorts were ‘confounded’ using a *p* < 0.01 loading threshold (critical value ≥±0.30). To minimize data loss, this significance threshold was systematically increased (Watts and Stenner 2005), and factor loadings of ≥±0.52 were deemed significant. Twenty-three Q-sorts loaded onto a factor, with the two remaining Q-sorts excluded.

The factors were highly correlated (Factors 1 and 2 = 0.79: Factors 1 and 3 = 0.71: Factor 2 and 3 = 0.75), indicating that although distinct aspects of opinion existed, there were substantial shared opinions.

Factor interpretations are presented by reporting the relevant statement and associated factor ranking. The presented statements used the term tic spectrum conditions (TSC)^1^ to refer to tic disorders. Quotes from post-sort questions are provided to supplement findings. Table 2 presents factor-specific participant information and Table 3 presents factor arrays.

**Table 2:** Participant information for each factor

Factor	Profession	Experience of parenting groups/interventions	Experience of working with tic disorders
1	Charity Worker	Yes, very frequently	Yes, very frequently, tic disorders training
1	Clinical Psychologist	Yes, very frequently, delivered IY	Yes, frequently, Neurology service
1	Clinical Psychologist	Yes, frequently, delivered parenting interventions	No
1	Clinical Psychologist	Yes, frequently, delivered IY	No
1	Trainee Clinical Psychologist	Yes, rarely, delivered IY	Yes, occasionally, one case
1	Trainee Clinical Psychologist	Yes, occasionally, delivered IY	No
1	Paediatric Neurologist	No	Yes, frequently, Neurology service
1	Primary Mental Health Worker	Yes, very frequently, delivered various parenting programmes	Yes, occasionally, several clinical cases
2	Charity Worker	No	Yes, frequently, support role.
2	Clinical Psychologist	Yes, very frequently	Yes, very frequently
2	Clinical Psychologist	Yes, very frequently, delivered ADHD parenting interventions	Yes, occasionally
2	Trainee Clinical Psychologist	No	Yes, frequently, research-based
2	Trainee Clinical Psychologist	Yes, occasionally, delivered ADHD parenting interventions	No
2	Trainee Clinical Psychologist	Yes, occasionally, delivered IY	No
2	Trainee Clinical Psychologist	Yes, frequently, delivered IY	No
2	Trainee Clinical Psychologist	No	Yes, occasionally
2	Trainee Psychotherapist	Yes, frequently, accredited IY group leader	No
2	Medic	Yes, frequently, delivered sleep-related programmes	Yes, frequently
3	Charity Worker	No	Yes, occasionally, delivered psychological intervention
3	Clinical Psychologist	Yes, frequently	No
3	Trainee Clinical Psychologist	Yes, occasionally, delivered IY	Yes, frequently, research-based
3	Research Assistant Psychologist	Yes, occasionally	Yes, very frequently, Neuropsychiatry/tic disorder clinic
3	Psychiatrist	Yes, very frequently	Yes, very frequently, Neuropsychiatry clinic

Two ‘confounded’ Q-sorts are excluded

*ADHD* attention deficit hyperactivity disorder, *IY* Incredible Years Programme (Webster-Stratton 2006)

**Table 3:** Factor Arrays showing Statements and Rankings

S	Statement	Factor		
		1	2	3
1^	Parents of children with TSC experience more stress than parents of children without TSC	0	−1	1
2	Helping parents to think about their own thoughts and feelings about their child’s difficulties is important in a parenting intervention	5	4	1*
3^	Parents own thoughts and feelings affect parenting behaviour	3	5	4
4	Giving parents time to talk about the worries they have about their child’s TSC is important in a parenting intervention	3	2	0
5	Learning skills to manage children’s anger is important in a parenting intervention for TSC	−1	1	0
6	Learning skills to manage children’s anxiety is important in a parenting intervention for TSC	2	4	0
7	Learning skills to develop a parent–child relationship through play, warmth, praise and attention is important in a parenting intervention for TSC	2	5	3
8^	Learning skills to give children positive attention, praise and rewards is important in a parenting intervention for TSC	3	3	4
9^	Learning skills in giving and enforcing clear commands to children is important in a parenting intervention for TSC	−1	−1	0
10^	Learning skills to pay less attention to children’s negative behaviours is important in a parenting intervention for TSC	1	1	−1
11^	Learning skills to apply consequences for negative behaviour (e.g., time out, grounding) is important in a parenting intervention for TSC	0	0	−1
12^	Learning skills to manage children’s mood is important in a parenting intervention for TSC	1	0	1
13^	Providing information about techniques to manage children’s tics (e.g., exposure response prevention, habit reversal training) is important in a parenting intervention for TSC	2	2	0
14	Helping parents accept and adjust to their child’s difficulties is important in a parenting intervention for TSC	6*	2	2
15	Providing education about tics is important in a parenting intervention for TSC	3	2	0
16	Providing information about medication (e.g., benefits, side effects) is important in a parenting intervention for TSC	2*	−3*	−1*
17	Helping parents to develop children’s social skills is important in a parenting intervention for TSC	1	1	3*
18	Making parents feel valued by providing a comfortable environment (e.g., snacks, breaks, resources) is important in a parenting intervention for TSC	1	3	0
19^	Parents would be worried that changing parenting techniques would make things worse	−2	−2	−2
20^	Inviting parents to attend a parenting intervention for TSC would make them feel criticised	−1	−2	−2
21^	If parents were given knowledge about psychological techniques (e.g., exposure response prevention, habit reversal) they would use these techniques to manage children’s tics	0	−1	−1
22	It would negatively affect the parent–child relationship if parents tried to change children’s tics using psychological techniques (e.g., exposure response prevention, habit reversal)	−3	−4	−6*
23^	Parents would be wary about a parenting intervention for TSC	−1	−2	−2
24^	The difficulties of children with TSC frequently change so a parenting intervention would not be effective over time	−4	−3	−3
25	Learning generalisable skills is important in a parenting intervention for TSC	0	0	4*
26^	TSC are biological in origin so a parenting intervention will have no effect	−5	−6	−5
27^	Parent interventions for TSC would be less effective than interventions that treat the child directly	−2	−2	−3
28^	Parents of children with and without TSC have similar needs so interventions just for children with TSC are unnecessary	−4	−3	−4
29^	Child and Adolescent Mental Health Services (CAMHS) should offer parenting interventions for TSC	1	1	1
30	Practical issues make it too difficult for parents to attend parenting interventions	−2	−1	1
31^	It is unreasonable to deliver an intervention through parents	−6	−5	−5
32	Parents would not complete homework as part of a parenting intervention because they are too stressed	−1	−2	−1
33^	Parents would not complete homework as part of a parenting intervention because they lack motivation	−3	−3	−3
34	Diagnosing TSC is a barrier to parents accessing interventions.	2*	−5*	−1*
35^	Parents would need repeated follow-ups to continue using the skills learned in a parenting intervention	0	0	−2
36^	Only a small number of people would need a parenting intervention for TSC	−2	−1	−2
37^	Parenting interventions for TSC are not a good use of NHS money	−5	−4	−4
38	Parents would only attend a parenting intervention if it involved other parents with children with TSC	0	0	−2*
39	Meeting other parents of children with TSC would increase parents worry about their own child	−3	−4*	−1
40^	Social support from other parents is an important benefit of a group-based parenting intervention	3	4	5
41^	Between six and ten parents in a group parenting intervention group is a good size	1	1	2
42	Parents should be offered a parenting intervention shortly after their child is first diagnosed with TSC	1	1	−1
43^	Parenting interventions are more appropriate for parents of younger children with TSC	−2	−1	−1
44	Transition to adolescence can be difficult so parenting interventions should be offered to parents of adolescents with TSC	0	1	2
45	Shorter, weekly meetings are better than longer, monthly meetings in a parenting intervention for TSC	−2*	0	0
46	Parents need to discuss their child’s difficulties on an individual basis	−1	−1	−3
47	If NHS resources are limited it is better for more parents to be seen in a group-based parenting intervention	−1*	3	1
48	Group-based parenting interventions for TSC are cost effective	1	0	6*
49	Family functioning is related to children’s adjustment and quality of life	4	2	3
50	Positive child-parent interactions are important for children’s adjustment and quality of life	4	6	4
51	Teaching parents the most effective parenting strategies will help to strengthen children’s social, emotional and academic competence	2	4	1
52	Parents are less likely to participate in group-based parenting interventions than individual parenting interventions	−1	−4*	−2
53	Children’s perception of their parent’s views towards their TSC is important	5	3	1*
54^	It is not children’s tics that cause most concern to parents, but common co-morbid conditions (e.g., anxiety, mood, anger, behavioural difficulties)	−1	−1	1
55	Parenting interventions for TSC should only be offered to parents of children with more severe tics	−4	−2	−4
56	The differences in children’s TSC related difficulties are a barrier to group-based parenting interventions	−3	−2	−4*
57	All main caregivers of a child need to attend a parenting intervention for it to be effective	−2	0*	−2
58^	Parents would accept and attend a parenting intervention for TSC	0	1	2
59	Professionals who run parenting groups for TSC must be experts in the treatment of tics	0	−2	−3
60^	It is important that parents have a positive relationship with the professionals that lead parenting interventions	1	3	3
61	The lack of research in parenting interventions for TSC is a barrier to treatment	−1	0	3*
62^	If a parenting intervention for TSC was in book form, professionals would be more likely to offer it	0	0	−1
63	Siblings of children with TSC would benefit from their parents attending a parenting intervention	2	1	0
64	It is important to consider parents’ cultural differences in a parenting intervention for TSC	3	1	3
65^	Helping parents to feel more in control of their child’s difficulties is an important outcome of parenting interventions for TSC	2	2	2
66	Changing children’s tics is an important outcome of parenting interventions for TSC	−3	−3	0*
67	Changing children’s common co-morbid difficulties (e.g., anxiety, anger, mood, behavioural difficulties) is an important outcome of parenting interventions for TSC	0	2	1
68	Helping parents to feel more positive about the future is an important outcome of parenting interventions for TSC	4	2	5
69	Parents prefer psychological interventions to medication for TSC	−2	−1	2*
70^	Medication is more effective than psychological interventions for TSC	−4	−3	−3
71^	Parenting interventions for TSC would be effective	1	3	2
72	Family members, friends, and teachers should be invited to attend parenting interventions for TSC	−3*	−1*	2*
73	A lack of training and knowledge about TSC is a barrier to non-specialist services offering parenting interventions for TSC	4*	0	0

^ = statistically consensus statements (*p* > 0.01). * = statistically distinguishing statement for factor (*p* < 0.01)

*TSC* tic spectrum condition/tic disorder, *NHS* national health service

All professionals agreed the importance of parenting practices on children’s well-being (e.g., Statement *50:‘Positive child*-*parent interactions are important for children’s adjustment and quality of life’; F1* = +*4, F2* = +*6, F3* = +*4),* and identified positive parenting skills as an intervention target (*s8:‘Learning skills to give children positive attention, praise and rewards is important in a parenting intervention for TSC’; F1* = +*3, F2* = +*3, F3* = +*4*). Professionals agreed with the importance of parents’ internal experiences in changing parenting practices (*s3:‘Parents own thoughts and feelings affect parenting behavior’; F1* = +*3, F2* = +*5, F3* = +*4*), such that an important intervention outcome was perceived parental control (*s65:‘Helping parents to feel more in control of their child’s difficulties is an important outcome of parenting interventions for TSC’; F1* = +*2, F2* = +*2, F3* = +*2*).

Professionals strongly endorsed the acceptability of parents as the agents for change, (*s31:‘It is unreasonable to deliver an intervention through parents’; F1* = −*6, F2* = −*5, F3* = −*5*) and (*s22:‘It would negatively affect the parent*–*child relationship if parents tried to change children’s tics using psychological techniques (e.g., exposure response prevention, habit reversal)’; F1* = −*3, F2* = −*4, F3* = −*6*). Parent-based motivation or worry were not seen as barriers (*s33: ‘Parents would not complete homework as part of a parenting intervention because they lack motivation’; F1* = −*3, F2* = −*3, F3* = −*3*) and (*s19:‘Parents would be worried that changing parenting techniques would make things worse’; F1* = −*2, F2* = −*2, F3* = −*2).* Group implementation was endorsed given social benefits, (*s40:‘Social support from other parents is an important benefit of a group*-*based parenting intervention’; F1* = +*3, F2* = +*4, F3* = +*5*).

In terms of effectiveness, professionals disagreed that biological or pharmacological approaches to tic disorders negates the effectiveness of psychological interventions (*s26:‘TSC are biological in origin so a parenting intervention will have no effect’; F1* = −*5, F2* = −*6, F3* = −*5*), and (*s70:‘Medication is more effective than psychological interventions for TSC’; F1* = −*4, F2* = −*3, F3* = −*3*). The effectiveness of parenting interventions was agreed, (*s71:‘Parenting interventions for TSC would be effective’; F1* = +*1, F2* = +*3, F3* = +*2*), (*s24:‘The difficulties of children with TSC frequently change so a parenting intervention would not be effective over time’; F1* = −*4, F2* = −*3, F3* =  −*3*) and (*s27:‘Parent interventions for TSC would be less effective than interventions that treat the child directly’; F1* = −*2, F2* = −*2, F3* = −*3*). All professionals endorsed a need and financial justification for tic-specific interventions (*s28:‘Parents of children with and without TSC have similar needs so interventions just for children with TSC are unnecessary’; F1* = −*4, F2* = −*3, F3* = −*4*), and (*s37:‘Parenting interventions for TSC are not a good use of NHS money’; F1* = −*5, F2* = −*4, F3* = −*4*).

### Distinguishing Factor Viewpoints

#### Factor 1: Reflecting, Accepting and Knowing

Eight professionals loaded onto Factor 1, explaining 25 % of the variance. As this factor represented the importance of parental cognitions and tic-specific education, it was termed ‘Reflecting, Accepting and Knowing’. Professionals included a range of professions, most had psychological training and reported considerable experience of parenting interventions. The focus on parental cognitions may therefore be underpinned by their systemic and reflective training backgrounds alongside common issues experienced through delivering parenting interventions across different populations.

Responses loading onto this factor particularly endorsed the importance of family environments and parental views on children’s well-being, (*s49:‘Family functioning is related to children’s adjustment and quality of life’;* +*4*) and (*s53:‘Children’s perception of their parent’s views towards their TSC is important’;* +*5*).

Professionals viewed parenting interventions as providing a reflective environment within which parents could explore and re-evaluate their cognitions to facilitate acceptance, adjustment and hope. Professionals strongly agreed with the following statements: (*s4:‘Giving parents time to talk about the worries they have about their child’s TSC is important in a parenting intervention’;* +*3*), (*s14:‘Helping parents accept and adjust to their child’s difficulties is important in a parenting intervention for TSC’;* +*6*), (*s2:‘Helping parents to think about their own thoughts and feelings about their child’s difficulties is important in a parenting intervention’;* +*5*) and (*s68:‘Helping parents to feel more positive about the future is an important outcome of parenting interventions for TSC’;* +*4*). Indeed, these parent-based outcomes were endorsed over tic modification (*s66:‘Changing children’s tics is an important outcome of parenting interventions for TSC’;* −*3*).

The need for providing specific tic-related knowledge to parents was agreed, (*s15:‘Providing education about tics is important in a parenting intervention for TSC’;* +*3*), and (*s16:‘Providing information about medication (e.g., benefits, side effects) is important in a parenting intervention for TSC’;* +*2*), and a lack of professional knowledge around tic disorders was perceived as an intervention obstacle (*s73:‘A lack of training and knowledge about TSC is a barrier to non*-*specialist services offering parenting interventions for TSC’;* +*4*).

In terms of attendants, delivering parenting interventions regardless of tic severity and to parents-only was endorsed, consistent with the advocated focus on parental cognitions, (*s55:‘Parenting interventions for TSC should only be offered to parents of children with more severe tics’;−4*) and (*72:‘Family members, friends, and teachers should be invited to attend parenting interventions for TSC’;* −*3*).

Comments provided by professionals loading onto Factor 1 highlighted the importance of parental cognitions:

“Parents who found it most hard to accept the disorder, and therefore their child, struggled the most and could not support their child.”

“Parental awareness, understanding, attitude, modelling and support are fundamental to a successful outcome in most cases. Regardless of tic severity.”

“Children pick [up] a lot on their parents perceptions and this will influence their self confidence and perception of themselves.”

#### Factor 2: Skilling-Up!

Ten participants loaded onto Factor 2, explaining 23 % of the variance. Factor 2, termed ‘Skilling-up!’, reflected a skills-based approach to parenting interventions. Professionals again encompassed a range of professions. Most had psychological backgrounds; however, many were still undertaking professional training. The value placed on delivering functional strategies to parents may therefore reflect their training stage, with perhaps greater focus on relaying learnt techniques.

Professionals strongly agreed the importance of effective parenting strategies on children’s well-being, (*s51:‘Teaching parents the most effective parenting strategies will help to strengthen children’s social, emotional and academic competence’;* +*4*). The value of parenting interventions was therefore viewed as providing practical skills to parents, (*s7:’Learning skills to develop a parent*–*child relationship through play, warmth, praise and attention is important in a parenting intervention for TSC’;* +*5*), and (*s6:‘Learning skills to manage children’s anxiety is important in a parenting intervention for TSC’;* +*4*). Professionals did endorse the importance of considering parents’ internal experiences in parenting interventions, (*s2:‘Helping parents to think about their own thoughts and feelings about their child’s difficulties is important in a parenting intervention’;* +*4*), perhaps given the perceived impact of internal experiences on parenting practices. These systemic intervention outcomes were again advocated over tic modification (*s66:‘Changing children’s tics is an important outcome of parenting interventions for TSC’;* −*3*).

In direct contrast to Factor 1, whilst professionals disagreed with the provision of medication information, (*s16:‘Providing information about medication (e.g., benefits, side effects) is important in a parenting intervention for TSC’;* −*3*), they did not deny the importance of acknowledging the medical underpinnings of the disorder, strongly disagreeing that (*s34:‘Diagnosing TSC is a barrier to parents accessing interventions’;* −*5*).

Responses also supported group-based delivery of interventions on resource and clinical grounds (*s47:‘If NHS resources are limited it is better for more parents to be seen in a group*-*based parenting intervention’;* +*3*), (*s39:‘Meeting other parents of children with TSC would increase parents worry about their own child’;* −*4*), and (*s52:‘Parents are less likely to participate in group*-*based parenting interventions than individual parenting interventions’;* −*4*).

Comments from professionals highlighted the perceived importance of parental strategies and group-based support:

“Likely to be beneficial both in terms of information and strategies for parents, and the social support parents may gain from a group.”

“A group has the potential to inform parents, provide social support, de-stigmatise Tourettes, and provide guidance.”

#### Factor 3: Generalisablility

Five participants loaded onto Factor 3, explaining 21 % of the variance. Factor 3, termed ‘Generalisability’, represented the universality of skills and attendants. Again the factor encompassed several professions, most with psychological training. Several professionals worked within specialist neuropsychiatry and tic disorder clinics and several held tic-related research roles. Consequently, the focus on generalisability may reflect increased awareness of the wider clinical needs of families alongside awareness of demands on specialist services.

Professionals strongly endorsed the importance of providing general skills, (*s25:‘Learning generalisable skills is important in a parenting intervention for TSC’;* +*4*). The importance of nurturing parental hope and children’s social skills were also advocated, (*s68:‘Helping parents to feel more positive about the future is an important outcome of parenting interventions for TSC’;* +*5*), and (*s17:‘Helping parents to develop children’s social skills is important in a parenting intervention for TSC’;* +*3*).

Consistent with the importance of generalisability, professionals disagreed that variability in children’s difficulties would be detrimental to interventions or that interventions should be limited to those with more severe tics, (*s56:‘The differences in children’s TSC related difficulties are a barrier to group*-*based parenting interventions’;* −*4*) and (*s55:‘Parenting interventions for TSC should only be offered to parents of children with more severe tics’;* −*4*). Professionals did not strongly advocate the need for tic-specific professional expertise or for individual interventions, (*s59:‘Professionals who run parenting groups for TSC must be experts in the treatment of tics’;* −*3*) and (*s46:‘Parents need to discuss their child’s difficulties on an individual basis’;* −*3*).

Group-based interventions were supported on financial grounds, (*s48:‘Group*-*based parenting interventions for TSC are cost effective’;* +*6*), and professionals encouraged wide attendance of significant others (*s72:‘Family members, friends, and teachers should be invited to attend parenting interventions for TSC’;* +*2*).

The current lack of research was, however, identified as an obstacle to implementation (*s61:‘The lack of research in parenting interventions for TSC is a barrier to treatment’;* +*3*), perhaps given professionals increased familiarity with research evidence in tic disorders.

Comments provided by professionals reflected the importance of general skills and research evidence:

“It can provide parents with generalisable skills and confidence in supporting their children and nurture family interactions and functioning.”

“They are enjoyable for the parents and they gain a lot [of] skills which they can use, either on their child with TS or on their siblings.”

“Unfortunately the evidence base is weak but clinically this a key component of good care.”

## Discussion

The study explored twenty-five professionals’ opinions on parenting interventions in tic disorders. Using Q-methodology, three factors were identified. Some shared views existed, with all factors endorsing a biopsychosocial approach, the importance of parenting practices for children’s well-being, and increased parental feelings of control. Given the range of participating professionals, this highlights the interdisciplinary recognition of systemic considerations and importance of multidisciplinary approaches within this population. Interventions were agreed to be needed, reasonable, effective, financially justifiable and well-received by parents across all factors, possibly reflecting practitioners increasing familiarity with popular parenting programmes (e.g., Webster-Stratton 2006).

Whilst shared general opinions were identified, factor-specific viewpoints also emerged. Factor 1 ‘Reflecting, Accepting and Knowing’ particularly endorsed the importance of providing a reflective environment to facilitate parental acceptance, adjustment and hope, alongside providing specialist tic-related information. Factor 2 ‘Skilling-up!’ particularly endorsed the importance of teaching parents effective parenting strategies, whereas Factor 3 ‘Generalisability’ particularly endorsed teaching generalisable skills.

### Methodological Limitations

The online Q-methodology paradigm enabled geographically dispersed professionals to participate. Although expression of opinions through researcher-generated statements can be criticised as restrictive and reductionist, this methodology opens participation to professionals who may not consider themselves able to freely generate extensive narratives around this topic, unlike qualitative approaches. This is important for tic disorders, given that prevalence and co-morbidities would suggest that children’s presentation within non-specialist services is likely. Surprisingly, whilst the majority of professionals indicated professional involvement with children with tic disorders and parents, only ten of the 25 professionals indicated frequent or very frequent professional experience of working with tic disorders. This suggests that some participants were not those who had particularly extensive professional experience of tic disorders; a scenario which could have potentially introduced bias into the sample. However, most professionals were from a clinical psychology background, possibly introducing some bias as a result of the over-representation of psychologists in recruitment sources. Whilst participants’ relative years of clinical experience can be inferred to some extent from their professional role (e.g., trainee clinical psychologist compared with clinical psychologist), collection of further demographic information regarding years of independent clinical practice, country of origin, age and gender would be helpful for future study replications. Further research should also extend recruitment sources and perhaps also consider professionals in areas such as education or social care.

A further potential criticism of the research is that some of the statements may have been highly endorsed as a result of their reflection of general best practice amongst child health care professionals. For example, one could assume that it is highly likely that a sample of child health care professionals will strongly endorse a statement such as s*50:‘Positive child*-*parent interactions are important for children’s adjustment and quality of life’.* Indeed, this statement was highly endorsed, and emerged as a shared opinion amongst factors (F1 = +4, F2 = +6, F3 = +4). The value of the current study, however, is that it innovatively demonstrates this assumed likelihood in an empirical manner. Indeed, the results may reflect the assumed current mindset of child health care professionals across many disorders, yet it is the first study to use such an approach in order to explore this mindset. The study also demonstrates for the first time that these shared and highly endorsed statements are viewed as applicable to tic disorders, highlighting the value of the transferable skills that general clinicians may already hold and could potentially use when working with tic disorder populations. Finally, whilst there are some shared opinions across professionals, there are distinct differences in the opinions that emerge and in the strength with which professionals endorse and prioritise statements. These nuisances offer important insights into viewpoints across professionals, and when considered in the context of the whole opinions that emerge for each factor, they provide considerably more than an assumed likelihood of strong agreement with individual statements.

### Clinical and Research Implications

Results hold obvious clinical implications for parenting interventions in tic disorders. A clear clinical justification for further development, implementation and evaluation of parenting interventions was identified. Lack of specialist knowledge and research evidence were endorsed as obstacles, identifying increased research and training needs in non-specialist services. Group interventions were endorsed as clinically appropriate and beneficial for financial, resource and social reasons. The study also provides guidance around general intervention content, identifying important components as teaching positive parenting skills, addressing parental cognitions and providing techniques to manage children’s anxiety and social skills. Surprisingly, intervention components directed at behavioural control were not strongly endorsed, despite the high co-morbidity and impact of behavioural difficulties on child and family functioning (Sukhodolsky et al. 2003).

In terms of these identified important components and their relevance to current clinical interventions in tic disorders, the single RCT by Scahill et al. (2006) was based on the Barkley ‘Defiant Children’ programme (1997). This structured programme included core skills such as providing positive reinforcement for appropriate behavior (token economies, positive attending), discouraging negative behaviour (consistent consequences, selective ignoring, time-out), and communication (communicating directions effectively) (Scahill et al. 2006). Whilst the Scahill et al. study was primarily oriented towards disruptive behaviours, it appears that the skills provided are aligned to some extent with those identified as important within the current study, such as teaching positive parenting skills, including praise and rewards. Furthermore, general parent training programmes such as the Incredible Years Programme (Webster-Stratton 2006) and Triple P (e.g., Sanders 1999) which also aim to provide techniques to promote positive parenting and child-parent interactions (e.g., play, quality time, limit setting, modelling, problem-solving) also provide skills advocated by the current study, and may thus offer some contribution to tic disorders. These programmes have also been successfully adapted and implemented in neurodevelopmental conditions (e.g., Leung et al. 2013; McIntyre 2008). The study also identified the general importance of addressing parental cognitions, thus considering cognitive components of parenting interventions may be appropriate, including possible acceptance and adjustment issues. Whilst the application of such components to tic disorders is limited, there is evidence of the application of such components in parenting interventions for children with neurodevelopmental disorders. For example, McIntyre’s (2008) adaptation of an Incredible Years parenting programme in developmental delay included discussions around the challenges and blessings of raising children with disabilities and Plant and Sanders’ (2007) enhanced Triple P Parenting Programme in developmental disabilities included content on grief and loss issues.

The study also however highlighted areas of contention among factors. Firstly, diagnosis was viewed as both a barrier (Factor 1) and facilitator (Factor 2) to interventions. The issues surrounding the complexity of diagnosis of tic disorders are well-documented (Robertson and Cavanna 2008) and divergence in professional opinion may reflect this wider debate. Similarly, the provision of medication information was endorsed (Factor 1) as well as contested (Factor 2). This highlights an important consideration and further research should explore parental opinions. Finally, attendance of significant others (e.g., teachers, friends) was contested (Factor 1) and endorsed (Factor 3). Previous parenting programmes in other populations have varied in terms of audience (e.g., Lee et al. 2012). Further research should determine the benefits and disadvantages in tic populations.

In conclusion, professionals generally agreed that interventions were theoretically and clinically justified but differences emerged in the advocated focus, barriers, and audience. Results hold clinical implications, and may aid development of a future programme, which could be implemented and evaluated within randomised controlled trials.

## Footnotes

1 This term is used because we believe that it is less stigmatising and reflects the complex range of symptoms.
